# Novel lncRNA UPLA1 mediates tumorigenesis and prognosis in lung adenocarcinoma

**DOI:** 10.1038/s41419-020-03198-y

**Published:** 2020-11-21

**Authors:** Xiaoyang Han, Hua Jiang, Jianni Qi, Jiamei Li, Jinghan Yang, Yingying Tian, Wei Li, Qi Jing, Chuanxi Wang

**Affiliations:** 1 Department of Oncology, Shandong Provincial Hospital, Cheeloo College of Medicine, Shandong University, Jinan, Shandong 250021 China; 2 Department of Thoracic Surgery, Shandong Provincial Hospital Affiliated to Shandong First Medical University, Jinan, Shandong 250021 China; 3 Department of Central Laboratory, Shandong Provincial Hospital Affiliated to Shandong First Medical University, Jinan, Shandong 250021 China; 4 Department of Pathology, Shandong Provincial Hospital Affiliated to Shandong First Medical University, Jinan, Shandong 250021 China; 5 Shandong Experimental High School, Jinan, Shandong 250001 China; 6 Department of Oncology, Shandong Provincial Hospital Affiliated to Shandong First Medical University, Jinan, Shandong 250021 China

**Keywords:** Non-small-cell lung cancer, Long non-coding RNAs

## Abstract

With the development of molecular biotechnology and sequencing techniques, long non-coding RNAs (lncRNAs) have been shown to play a vital role in a variety of cancers including lung cancer. In our previous study, we used RNA sequencing and high-content screening proliferation screening data to identify lncRNAs that were significantly associated with tumour biological functions such as LINC01426. Herein, based on previous work, we report a novel lncRNA UPLA1 (upregulation promoting LUAD-associated transcript-1), which has not been explored or reported in any previous studies. Our results showed that UPLA1 is highly expressed and regulates important biological functions in lung adenocarcinoma. In vitro experiments revealed that UPLA1 promoted the migration, invasion, and proliferation abilities, and is related to cell cycle arrest, in lung adenocarcinoma cells. Moreover, the upregulation of UPLA1 significantly improved the growth of tumours in vivo. We identified that UPLA1 was mainly located in the nucleus using fluorescence in situ hybridisation, and that it promoted Wnt/β-catenin signalling by binding to desmoplakin using RNA pulldown assay and mass spectrometry. Additionally, luciferase reporter assay revealed that YY1 is the transcription factor of UPLA1 and suppressed the expression of UPLA1 as a transcriptional inhibitor. This finding provides important evidence regarding the two roles of YY1 in cancer. Furthermore, in situ hybridisation assay results showed that UPLA1 was closely related to the prognosis and tumour, node, metastasis (TNM) stage of lung adenocarcinoma. In summary, our results suggest that the novel lncRNA UPLA1 promotes the progression of lung adenocarcinoma and may be used as a prognostic marker, and thus, has considerable clinical significance.

## Introduction

Cancer is a primary public health problem and a major cause of death worldwide, and lung cancer is the principal cause of oncogenic mortality in both men and women^1,2^. Moreover, lung adenocarcinoma (LUAD) is the most common type of lung cancer, accounting for more than 40% of lung cancers, 60% of non-small cell lung cancers (NSCLC), and more than 70% of surgically resected cases^3^. Although there have been advances in radiotherapy, chemotherapy, and immunotherapy for lung cancer, its 5-year survival rate is still not satisfactory^4^. Genetic alteration is considered to be an important cause of tumorigenesis^5^.

Based on efforts to regulate tumorigenesis at the genetic level, recent advances and epigenetic insights have suggested that long non-coding RNAs (lncRNAs) may be important tumour regulatory factors^6,7^. LncRNAs are transcripts of more than 200 nucleotides that do not encode proteins. The number of lncRNAs vary widely in mammals, ranging from less than 20,000 to over 100,000 in humans^8^. Several previous studies have identified important roles of lncRNAs in essential biological processes such as cellular proliferation, migration, and maintenance of pluripotency of stem cells, all of which are essential for tumour progression^9,10^. Thus, as regulators of LUAD pathogenesis, lncRNAs are considered as potential targets at the genetic level for cancer therapy and monitoring. With increasing research, lncRNAs have assumed extensive clinical significance. Some lncRNAs, such as SOX2OT and ANRIL, are valuable for the diagnosis and prognosis of NSCLC^11^, while others, such as AK126698, AFAP1-AS1, and H19, are associated with drug resistance and prognosis^12–14^. Although research has indicated that lncRNAs play an important role in cancer, many that can act as tumorigenesis and prognosis markers have not been found.

Previously, LUAD and normal tissue samples were used to reveal differentially expressed lncRNAs using RNA sequencing data analysis. Subsequently, high-content screening proliferation assay was used to screen 18 lncRNAs with apparently abnormal differences and we identified lncRNAs with oncogenic functions such as LINC01426^7^. Here, we identified a novel lncRNA termed upregulation promoting LUAD-associated transcript-1 (UPLA1) based on expression differences in carcinoma and normal tissues. Its low expression after knockdown in the high-content screening proliferation assay revealed that it may act as an oncogene. Our results confirmed this hypothesis, as it was highly expressed in LUAD tissues and was found to be clinically associated with advanced stage and poor prognosis in patients with LUAD. Moreover, we demonstrated that the inhibition of UPLA1 significantly affected cell proliferation, migration, and apoptosis in vitro and in vivo. Additionally, we identified that the lncRNA UPLA1 directly binds to desmoplakin (DSP) and promotes the activity of Wnt/β-catenin using RNA pulldown and western blotting assays. We also found that YY1 was a transcription factor for UPLA1 and inversely regulated UPLA1 as a tumour suppressor.

In summary, we found that lncRNA UPLA1 was regulated by YY1 and promoted Wnt/β-catenin signalling by binding to DSP, thus leading to tumour progression in LUAD. It was markedly associated with tumour, node, metastasis (TNM) stage, poor survival, and prognosis in patients with LUAD, and may be a novel marker and target for LUAD treatment.

## Materials and methods

### Cell culture

The LUAD cell lines A549 and H1299 were acquired from ScienCell Research Laboratories (San Diego, CA, USA). A549 and H1299 cells were cultured in Ham’s F-12K (Gibco, Grand Island, NY, USA) and RPMI-1640 (Gibco) media, respectively. Both media contained 10% HyClone Fetal Bovine Serum (Gibco) and 1% Penicillin-Streptomycin solution (Sigma, St. Louis, MO, USA). The cells were maintained at 37 °C in a 5% CO_2_ incubator.

### Clinical tissues

Seventy-eight pairs of surgically resected LUAD and matched adjacent normal tissue samples were obtained from patients at Shandong Provincial Hospital affiliated to Shandong University, who were followed-up till July 2012. None of the patients received preoperative chemotherapy or radiotherapy. The tissue samples were stored at −80 °C. This study was overseen and approved by the Committee for Ethical Review of Research involving Human Subjects at Shandong Provincial Hospital (Approval no. 2018-102).

### RNA sequencing and data analysis

RNA sequencing and data analysis was conducted in accordance with a previously established protocol^7^.

### RNA isolation and qRT-PCR analysis

RNA isolation and quantitative reverse transcription polymerase chain reaction (qRT-PCR) assays were conducted in accordance with a previous protocol^7^. The 2^−^^ΔΔCt^ method was used to calculate the relative gene expression normalised using glyceraldehyde 3-phosphate dehydrogenase. The primers for qRT-PCR of lncRNA UPLA1 were 5′‐AGGATGCTGTGGGAAGAGTG‐3′ (forward) and 5′‐GAGTTATGAAAGCGGAGTTGC‐3′ (reverse). The primers for YY1 were 5′‐AGATCCCAAACAACTGGCAGA‐3′ (forward) and 5′‐TCTTTGTGCAGCCTTTATGAGG-3′ (reverse). The primers for DSP were 5′‐TGAAAACCTGCTGAAAGCGTC‐3′ (forward) and 5′-GCCTCCTGTTTCTGAGCGAT-3′ (reverse). The primers for glyceraldehyde 3-phosphate dehydrogenase were 5′‐TGACTTCAACAGCGACACCCA‐3′ (forward) and 5′‐CACCCTGTTGCTGTAGCCAAA‐3′ (reverse). The primers for RT-PCR of UPLA1 were 5′-CATTTGTTTCCTTTTACTTTTTTAA-3′ (forward) and 5′-ATGAAAGAGGGAGCAGGAGGC-3′ (reverse).

### Western blotting

Western blotting was conducted in accordance with a previous protocol^7^. The primary antibodies (1:1000) used were DSP (Santa Cruz Biotechnology, Santa Cruz, CA, USA), β-catenin (Santa Cruz Biotechnology), β-actin (Cell Signaling Technology, Danvers, MA, USA), and histone H3 (Cell Signaling Technology).

### Cell transfection

The recombinant lentivirus of lncRNA UPLA1 (short hairpin (sh)-UPLA1) knockout and negative control (sh−Ctrl) were synthesised by GeneChem (Shanghai, China). The small interfering RNAs targeting DSP and YY1 and matched negative controls were synthesised by Genomeditech (Shanghai, China). The plasmids of lncRNA UPLA1 and YY1 were purchased from BioSun (Shanghai, China). The cell transfection was performed using Lipofectamine 3000 (Invitrogen, Waltham, MA, USA), according to manufacturer’s instructions. QRT-PCR was performed to evaluate the transfection efficiency. Control groups: sh-Ctrl, pcDNA-NC, and si-NC; experiment groups: sh-UPLA1, pcDNA-YY1, si-YY1, and si-DSP.

### MTT and colony formation assays

MTT (3-(4,5-dimethylthiazol-2-yl)-2,5-diphenyl tetrazolium bromide) and colony formation assays were conducted in accordance with a previously established protocol^7^.

### Flow cytometric analysis

Flow cytometric assay was performed in accordance with a previously established protocol^7^. The cells and their populations were analysed using FlowSight Imaging Flow Cytometer (Merck MilliporeShanghai, China) to estimate the number of cells in the G0/G1, S, and G2/M phases. The data were analysed using the CellQuest Pro software (BD Biosciences, USA). Every experiment was conducted at least thrice.

### Transwell migration, invasion, and wound-healing assays

Transwell migration, invasion, and wound-healing assays were conducted in accordance with a previous protocol^7^.

### RNA-fluorescence in situ hybridisation (FISH)

FISH was performed using Ribo FISH Kit (RiboBio, Guangdong, China) according to the manufacturer’s instructions^7^. The subcellular location of UPLA1 was detected using confocal microscopy.

### RNA pulldown assay

RNA sequences were obtained using the FastDigest XhoI Kit (Thermo Fisher Scientific, Waltham, MA, USA) and T7 MEGAscript Kit (Invitrogen), while antisense sequences were obtained using the FastDigest HindIII Kit (Thermo Fisher Scientific) and SP6 MEGAscript Kit (Invitrogen). The transcription reactions were allowed to proceed for 3 h at 37 °C.

Pierce RNA 3′ End Desthiobiotinylation Kit (Thermo Fisher Scientific) and Pierce Magnetic RNA-Protein Pull-Down Kit (Thermo Fisher Scientific) were used for RNA pulldown, following the manufacturer’s instructions with minor modifications. The samples to be tested were identified by mass spectrometry performed by the Beijing Institute of Animal Husbandry and Veterinary Medicine.

The full-length sequence of lncRNA UPLA1 was amplified and cloned into pcDNA3.0.

### Luciferase reporter assay

The luciferase reporter assay was performed according to the manufacturer’s instructions (Genomeditech). The wild and mutant types of UPLA1 promoter sequences were synthesised and constructed into the pGL3 plasmid. The constructed reporter plasmids were used to transfect HEK-293, UPLA1-WT, UPLA1-MT-site1, UPLA1-MT-site2, UPLA1-MT-site3, UPLA1-MT-site4, pcDNA3.1-NC, and pcDNA3.1-YY1 (mutation sites with the four highest scores predicted by the JASPAR database). The multifunctional microplate reader Infinite M1000 (Tecan, Männedorf, Switzerland) was used to quantify the luminescent signal, according to the manufacturer’s instructions. The detected relative luciferase activity was normalised to the *Renilla* luciferase activity.

### ISH assay

The ISH probe of lncRNA UPLA1 was designed according to the lncRNA UPLA1 sequence from UCSC (access ID: ENST00000578774.1). The probe was modified using antisense oligodeoxynucleotides and the sequence was 5′-TCATACTGGTCTCATCGCCTAA-3′. All paraffin-embedded tissue samples were tested following the manufacturer’s instructions. Briefly, the paraffin-embedded tissue sections were dewaxed and rehydrated. After the hydroperoxidase activity was blocked, the slices were then digested with protease K and rinsed with 0.5 M phosphate-buffered saline. Subsequently, the hybridisation buffer containing the probes was added and incubated at 65 °C for 12–16 h. The coverslip was rinsed with phosphate-buffered saline and 4% paraformaldehyde was added for fixation. Positive cells exhibited blue-violet granules in the cytoplasm or membrane. The sample was labelled as positive if more than 40% of the cells were positive. The positive rate of all samples was calculated.

### Establishment of an in vivo model

Four-to-five-week-old BALB/c nude mice were divided into two groups. A549/sh-Ctrl and A549/sh-UPLA1 were prepared as cell suspensions with 3 × 10^6^ cells/100 µL (phosphate-buffered saline : basement membrane = 1:1) (Basement Membrane; Corning, NY, USA). The suspensions were administered subcutaneously to nude mice and the tumour growth was observed regularly. After about 40 days, tumour-bearing mice were anesthetised and photographed. Subsequently, tumour peeling was performed. Tumours were weighed and fixed in paraformaldehyde. Ethical approval for the animal experiments was obtained from Shandong Provincial Hospital for Animal Experimentation (No. 2019122).

### Statistical analysis

All statistical analysis was performed using SPSS version 25.0, Microsoft Excel version 16.30, and GraphPad Prism version 7.0. Student’s *t*-test was used to compare the differences and one-way analysis of variance was used to compare the mean values between groups. Multiple comparisons between the groups was performed using the Student-Newman-Keuls method. Chi-squared test and Spearman rank correlation analysis were used to analyse the associations between lncRNA UPLA1 expression and clinicopathological variables. Survival was estimated using the Kaplan-Meier log-rank method and independent predictors were estimated using Cox univariate and multivariate regression analyses. *P* < 0.05 was considered statistically significant.

## Results

### LncRNA UPLA1 is expressed in LUAD

**Fig. 1:**
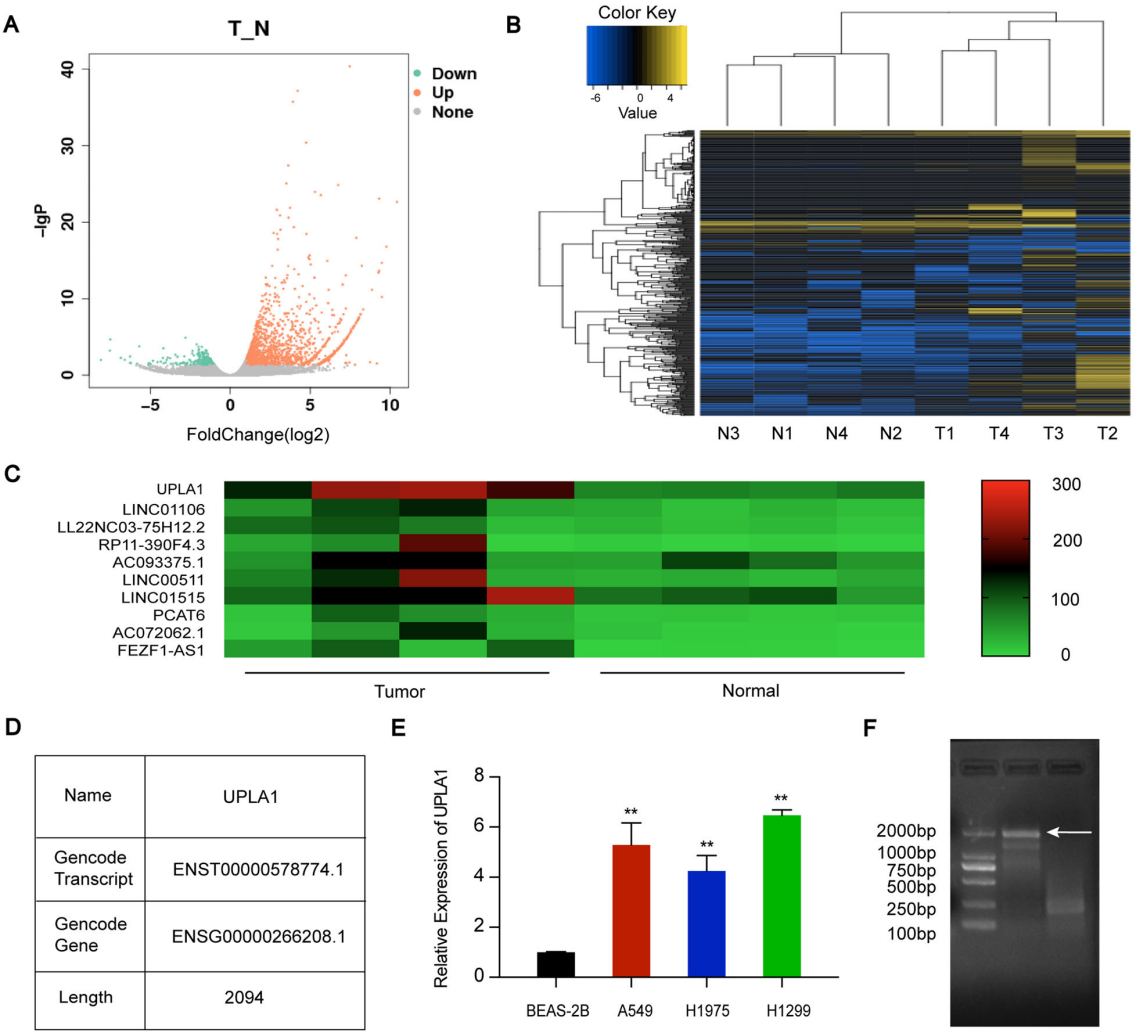
High-lncRNA UPLA1 expression in LUAD tissues and cell lines. **A** Volcanic map of lncRNA expression in LUAD and matched normal tissues. Green and orange symbols indicate significantly down- and upregulated lncRNAs in LUAD, respectively. **B** Cluster map showing differentially expressed genes in each sample and overall sample clustering. Blue and yellow indicate lower and higher expression, respectively. **C** Heat map of upregulated lncRNAs. **D** Information on UPLA1 in the UCSC database. **E** QRT-PCR analysis of UPLA1 expression in A549, H1299, and H1975 cells compared to that in BEAS-2B cells. **F** Full-length UPLA1 sequence on agarose gel electrophoresis. All values are expressed as mean ± SD (**P < 0.01 by *t*-test).

The analysis of four pairs of LUAD and normal tissue samples revealed differentially expressed lncRNAs (Fig. 1A). As shown in Fig. 1B, some lncRNAs in the LUAD samples showed highly differential expression compared to the matched normal tissue samples (*P* < 0.05). A novel lncRNA UPLA1 with upregulated expression, located on chromosome 17: 40 360 655–40 364 693 was discovered (data from UCSC and Ensembl databases; Fig. 1C, D). On this basis, we used RT-PCR to identify the accuracy of the length of UPLA1 (2 094 bp; Fig. 1F).

UPLA1 expression was examined in LUAD cell lines and normal lung bronchial epithelial cells using qRT-PCR, and the results showed that UPLA1 was highly expressed in A549, H1299, and H1975 cells compared to BEAS-2B cells (Fig. 1E). Therefore, A549 and H1299 cell lines were selected for further investigation.

### Effects of IncRNA UPLA1 knockdown

**Fig. 2:**
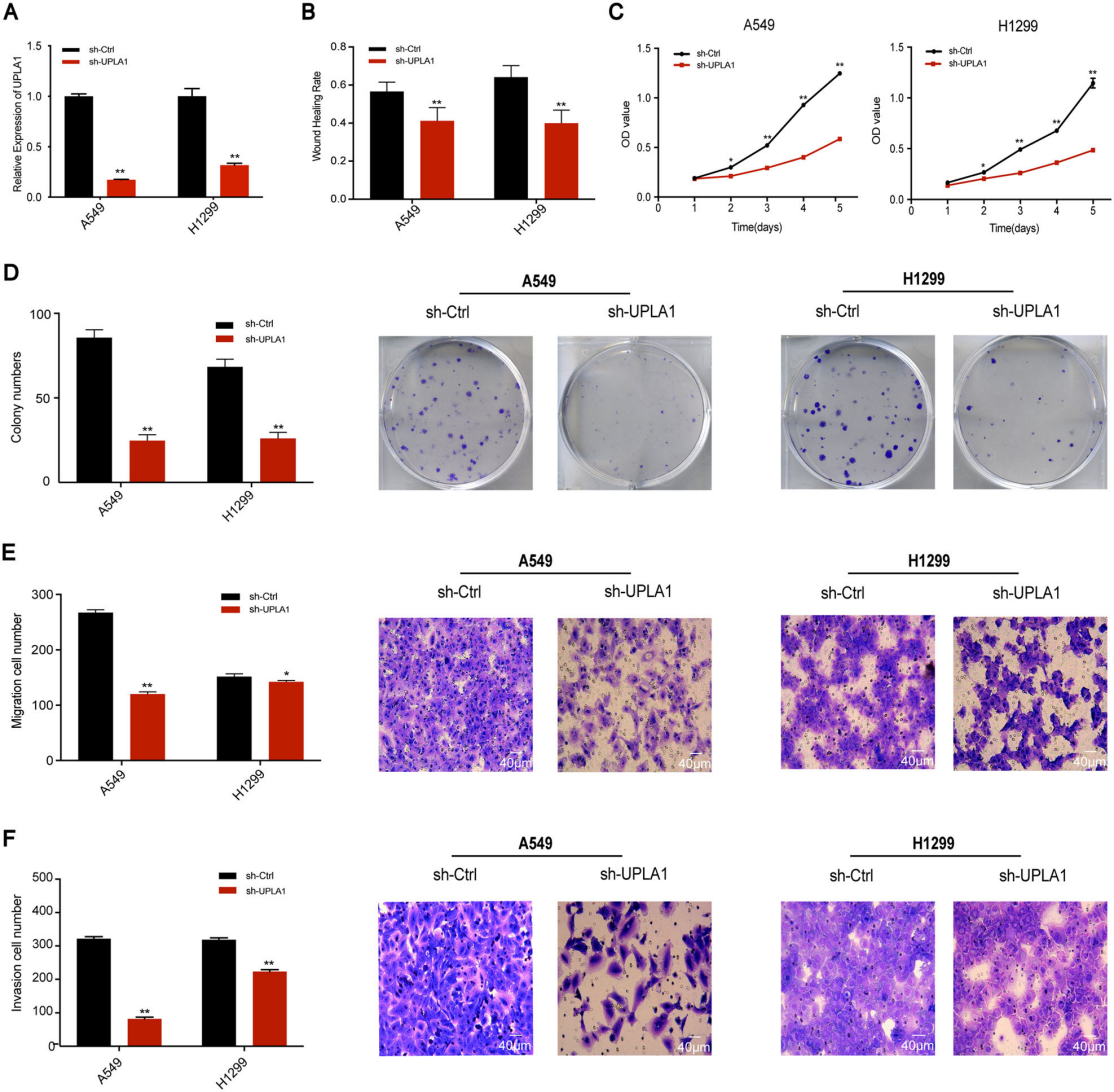
UPLA1 promotes LUAD cellular growth and viability in vitro. **A** Effects of UPLA1 knockdown were evaluated using qRT-PCR. **B** Effects on migration were analysed using the wound-healing assay. **C**, **D** MTT and colony formation assay results suggest that cell viability and proliferation were inhibited in A549 and H1299 cells. **E**, **F** Transwell and invasion assays demonstrated that migration and invasion were inhibited after UPLA1 knockdown in A549 and H1299 cells. All values are expressed as mean±SD (*P < 0.05, **P < 0.01 by *t*-test).

UPLA1 was stably knocked down in A549 and H1299 cells via transfection of sh-UPLA1 and sh-Ctrl (NC group), and the transfection efficiency was confirmed by qRT-PCR (Fig. 2A). The wound-healing assay revealed that the migration ability of the two cell lines in the sh-UPLA1 group reduced at 24 h post-wounding compared to the NC group (Fig. 2B). The MTT assay showed that compared with the NC group, UPLA1 downregulation significantly reduced the viability of A549 and H1299 cells (Fig. 2C). Additionally, the colony formation capability of the sh-UPLA1 group was lower than that of the NC group (Fig. 2D). Transwell migration and invasion assays demonstrated that UPLA1 downregulation led to a significant reduction in the migration and invasive abilities of the two cell lines (Fig. 2E, F). These results showed that lncRNA UPLA1 can promote the proliferation, migration, and metastasis of A549 and H1299 cells.

### Effects on cell cycle arrest/apoptosis

**Fig. 3:**
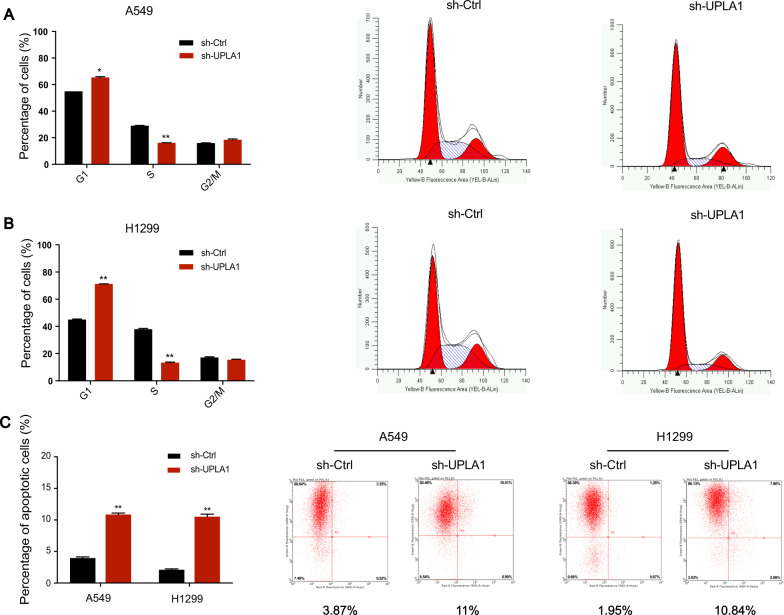
LncRNA UPLA1 affects cell cycle and apoptosis in A549 and H1299 cells. **A**, **B** Cell cycle stages of A549 and H1299 cell lines were evaluated using flow cytometry. **C** Effects of UPLA1 on apoptosis were analysed using flow cytometry. All values are expressed as mean ± SD (*P < 0.05, **P < 0.01 by *t*-test).

Flow cytometric analysis of A549 cells showed a significant increase in the percentage of cells in the G0/G1 phase but decrease in those in the S phase in the sh-UPLA1 group compared to that in the NC group (Fig. 3A). Analysis of H1299 cells revealed similar results, with a reduction in cells in the S phase and increase in cells in the G0/G1 phase in the sh-UPLA1 group (Fig. 3B).

Flow cytometric analysis for apoptosis indicated that the downregulation of UPLA1 had a significant impact on the percentage of apoptotic A549 and H1299 cells compared to the NC group (Fig. 3C). These results show that lncRNA UPLA1 plays an important role in cellular biological functions including cell cycle progression and apoptosis in LUAD.

### Subcellular location of UPLA1

Localisation of lncRNAs in the nucleus or cytoplasm is closely related to their function. Our results showed that UPLA1 was mainly localised to the nucleus in H1299 and A549 cells.

**Fig. 4:**
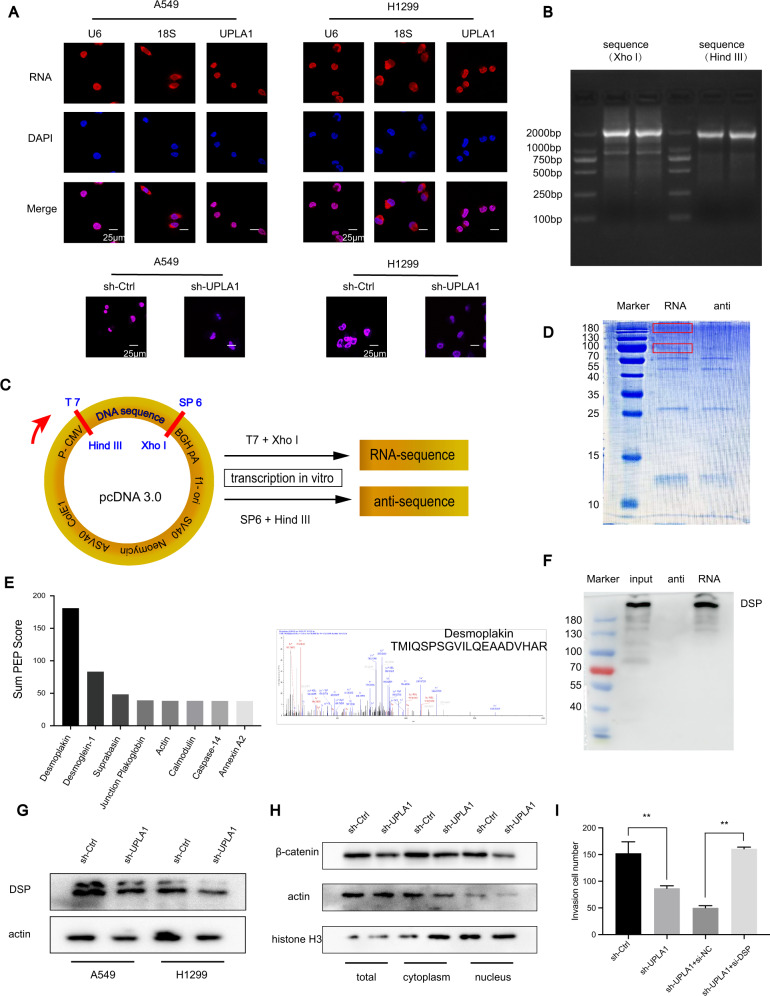
LncRNA UPLA1 is predominantly located in the cell nucleus and binds to DSP. **A** Subcellular location of UPLA1 was detected by FISH assay in A549 and H1299 cells after transfection. **B** The plasmid was treated with FastDigest enzymes XhoI and HindIII, and the sequences obtained were used as a PCR template. **C** LncRNA UPLA1 was inserted into pcDNA 3.0 vector. **D** Proteins obtained by RNA pulldown and results of Coomassie blue staining. **E** The top ten proteins obtained by RNA pulldown were subjected to mass spectrometry and DSP was identified as the main UPLA1-interacting protein. **F** Proteins obtained from RNA pulldown were verified by western blotting, which showed that UPLA1 binds to DSP. **G** Expression of DSP by western blotting in A549/sh-Ctrl and sh-UPLA1. **H** Western blotting demonstrated that Wnt/β-catenin signalling decreased after lncRNA UPLA1 knockdown in total cell, cytoplasmic, and nuclear proteins, and was significantly inhibited in total cell and nuclear proteins. **I** Rescue assay showed that the invasive ability of the sh-UPLA1 + si-DSP group returned to the level of that in the sh-Ctrl group. All values are expressed as mean ± SD (**P < 0.01 by *t*-test).

To determine the accuracy of the probe, confocal microscopy was performed after transfection, and these results showed that the fluorescence of sh-UPLA1 was markedly weakened compared to the NC group (Fig. 4A).

### UPLA1 binds to DSP

Since lncRNA UPLA1 was found to act as a tumour promoter and was mainly located in the nucleus, we examined its mechanism by performing RNA pulldown assay using biotin-labelled RNA to identify proteins associated with lncRNA UPLA1 in the A549 cell line. Subsequently, the proteins obtained from sodium dodecyl sulphate polyacrylamide gel electrophoresis were stained with Coomassie blue (Fig. 4D). The remaining bands specific to lncRNA UPLA1 were excised and subjected to mass spectrometry, which identified DSP as an RNA-binding protein (Fig. 4E). Then, we used western blotting and another independent RNA pulldown assay to verify the UPLA1-DSP interaction (Fig. 4F). We examined the effect of DSP expression by qRT-PCR and western blotting, and the results indicated that lncRNA UPLA1 knockdown in A549 and H1299 cells had no significant effect on *DSP* mRNA (data not shown) and protein levels (Fig. 4G).

Previous studies have demonstrated that the low expression of desmosomes was associated with invasion and migration behaviour of tumour cells^15,16^. A reduction in desmosomes has been identified in breast, colorectal, and pancreatic cancers among others^17–19^. As an important component of desmosomal plaque proteins, the function of DSP was verified in lung cancer^20^. Given that studies have suggested that DSP is located in the nucleus^21^, its downregulation was found to inhibit the proliferation, migration, and invasion in the tumour cells. Furthermore, DSP plays a key role in Wnt/β-catenin signalling and acts as a tumour suppressor in NSCLC ^20^. These functions were consistent with the results in our study (Supplementary Fig. A–E).

As DSP has an obvious inhibitory effect on Wnt/β-catenin signalling, we noted that the knockdown of lncRNA UPLA1 significantly inhibited Wnt/β-catenin signalling (Fig. 4H) and used an invasion assay as a rescue assay (Fig. 4I). We inferred that the reduction of DSP was not caused by the overexpression of lncRNA UPLA1 in LUAD. Notably, UPLA1 could inhibit the tumour suppressor activity of DSP after binding to DSP and promote Wnt/β-catenin signalling.

### LncRNA UPLA1 is regulated by YY1

**Fig. 5:**
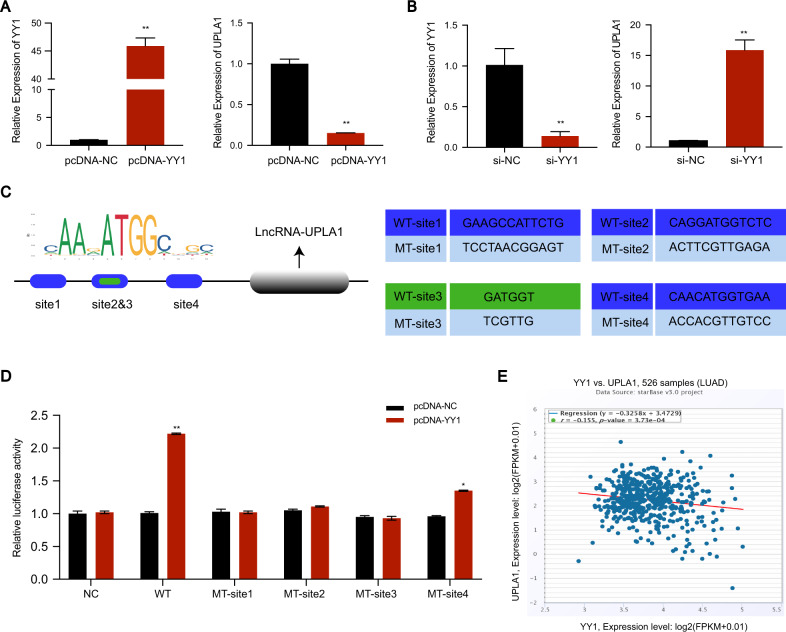
YY1 is the transcription factor of lncRNA UPLA1 and inversely regulates its expression. **A** QRT-PCR shows that lncRNA UPLA1 expression was significantly inhibited by overexpression of YY1. **B** QRT-PCR shows that lncRNA UPLA1 expression increased significantly after YY1 knockdown. **C** The binding site of YY1 to the promoter region of UPLA1, and the sequence of the wild-type and mutant-type YY1 is shown. **D** Luciferase reporter assay demonstrates that luciferase activity decreased significantly after mutation in four sites. **E** Co-expression relationship between YY1 and UPLA1 shows negative correlation (data obtained from StarBase). All values are expressed as mean ± SD (*P < 0.05, **P < 0.01 by *t*-test).

The promoter sequence of UPLA1 was obtained from the UCSC database and the transcription factor YY1 was selected from the PROMO and JASPAR databases. Subsequently, we used small interfering-YY1 and pcDNA-YY1 to transfect A549 cells. The overexpression of YY1 led to a remarkable inhibition of UPLA1 expression (Fig. 5A), and the knockdown of YY1 led to the significant upregulation of UPLA1 expression (Fig. 5B). Next, we screened the first four binding sites (site 1: 280–291, site 2: 633–644, site 3: 636–641, and site 4: 912–923) that were found to be the most reliable sites using the JASPAR database, and mutated them using the luciferase reporting assay for verification (Fig. 5C). Interestingly, our results showed that all the four sites were YY1 binding sites. MT-site 1 and MT-sites 2 and 3 exhibited the strongest binding ability, and the luciferase activity was not significantly different from the matched NC group. We speculated that there was a synergistic effect between site 1 and sites 2 and 3, whereas site 4 might be a single binding site and was not affected by site 1 and sites 2 and 3 (Fig. 5D). The co-expression models of YY1 and UPLA1 in StarBase showed a negative correlation (Fig. 5E), which is consistent with our findings. Consequently, we identified that YY1 is the transcription factor of UPLA1 and inversely regulates UPLA1 expression.

### Effects of overexpression of UPLA1

**Fig. 6:**
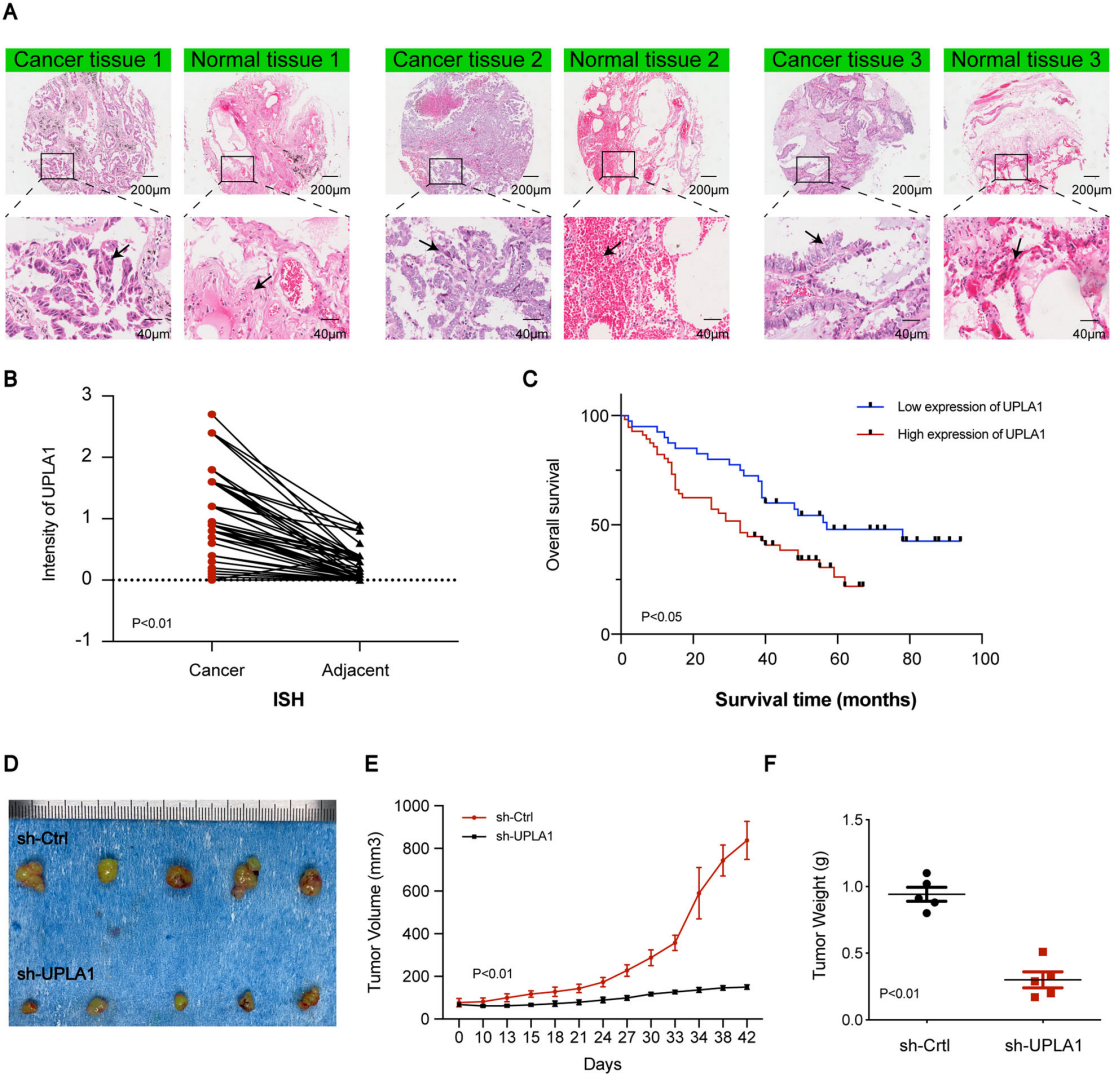
LncRNA UPLA1 expression correlates with poor prognosis in LUAD. **A** In situ hybridisation assay showing UPLA1 expression in 78 LUAD and matched normal tissues (as shown by the arrow, the stronger blue-violet intensity reflects higher expression of UPLA1). **B** The ordinate represents the expression of UPLA1 (*P* < 0.01). **C** Survival time of patients with LUAD after surgery in the high-UPLA1 expression group was remarkably shorter (*P* < 0.05). **D** In nude mice, tumours in the sh-Ctrl group were larger than those in the sh-UPLA1 group. **E** There was a significant difference in the tumour volumes between the sh-Ctrl and sh-UPLA1 groups (*P* < 0.01). **F** Tumours in the sh-UPLA1 group weighed lesser than those in the sh-Ctrl group (*P* < 0.01). All values are expressed as mean ± SD (by *t*-test, Kaplan-Meier analysis and log-rank test).

The UPLA1 level in lung cancer tissue was found to be higher than that in ISH-based matched normal lung tissue (Fig. 6A, B). Patients with LUAD were graded according to the UPLA1 expression and different clinicopathological features (sex, grade, age, T stage, N stage, M stage, and TNM stage). Chi-squared test indicated that UPLA1 was highly expressed in patients with LUAD and Spearman analysis showed that lncRNA UPLA1 expression was related to the TNM stage (*P* < 0.001), N stage (*P* < 0.05), and T stage (*P* < 0.01; Tables 1 and 2). Kaplan-Meier analysis and log-rank test demonstrated that UPLA1 expression correlated with overall survival (*P* < 0.05; Fig. 6C). The univariate Cox regression analysis revealed that overall survival was correlated with the expression of lncRNA UPLA1, age, T stage, N stage, and TNM stage, while multivariate analysis confirmed that survival was associated with the TNM stage (*P* < 0.05; Supplementary Table). Therefore, lncRNA UPLA1 expression may be used as an independent prognostic factor in patients with LUAD. Further, A549/sh-Ctrl and A549/sh-UPLA1 were subcutaneously injected into nude mice, the volume and weight of the tumours decreased significantly after knockdown of lncRNA UPLA1 (*P* < 0.01; Fig. 6D–F).

**Table 1:** Differential expression of UPLA1 in cancerous and lung tissues.

	*n*	Expression		*Χ*^2^ value	*P* value
		High(%)	Low(%)		
Lung adenocarcinoma	78	46	32	51.92151	5.78E-13
Lung tissues	78	4	74		

*Statistically significant (*P* < 0.05).

**Table 2:** Correlation between UPLA1 expression and clinicopathological characteristics.

Variables	UPLA1 expression		Total	*χ*2 value	*P* value
	Low	High			
Age(year)				1.818	0.178
<60	22	23	45		
å =60	18	33	51		
T stage				7.554	0.006
T1/T2	34	36	70		
T3/T4	4	20	24		
Sex				0.392	0.531
Female	16	26	42		
Male	24	30	54		
TNM stage				14.890	0.000
Ι/II	36	29	65		
III/IV	4	26	30		
N stage				4.176	0.041
N0	23	20	43		
N1/N2/N3	17	35	52		
M stage				0.722	0.396
M0	40	55	95		
M1	0	1	1		
Grade				0.000	1.000
I/II	25	35	60		
III	15	21	36		

*Statistically significant (*P* < 0.05).

In summary, the expression of lncRNA UPLA1 was significantly correlated with the TNM stage, survival time, and prognosis in patients with LUAD.

## Discussion

In this study, knockdown of lncRNA UPLA1 significantly suppressed tumour cell proliferation, migration, and invasion, and affected the cell cycle. Mechanically, RNA pulldown demonstrated that lncRNA UPLA1 principally binds to DSP, and western blotting showed that it promotes Wnt/β-catenin signalling. For RNA pulldown, full-length UPLA1 and its antisense sequence was inserted into the pcDNA 3.0 vector, to verify the accuracy of the plasmid and endonuclease, the plasmid was treated with FastDigest XhoI and HindIII, and the cut sequences were used as a template for PCR (Fig. 4B). Digested using FastDigest XhoI, and transcribed in vitro using the T7 promoter to obtain the full-length RNA sequence. Similarly, the full-length of antisense sequence was obtained using FastDigest HindIII and in vitro transcription with the SP6 promoter (Fig. 4C). However, after sodium dodecyl sulphate-polyacrylamide gel electrophoresis and cutting of the gel to recover the proteins obtained in RNA pulldown, many proteins were lost. Therefore, the second experiment wherein a portion of the protein samples was processed by electrophoresis to identify the molecular weight distribution of specific proteins compared to that of the marker, and the remaining parts were identified using MS directly and this gave us favourable results.

Although there is abundant evidence to support the role of YY1 as a tumour promoter, recent reports have shown that YY1 also has a tumour inhibitory effect. The mechanism by which YY1 produces opposite effects on tumour growth and inhibition, and acts as both transcription activator and inhibitor is still unknown^22^. For example, YY1 acts as an activator to promote lncRNA MCM3AP-AS1 in LUAD^23^. Our study revealed that YY1 inversely regulated the expression of lncRNA UPLA1 using qRT-PCR and luciferase reporter assays. Therefore, we found that YY1 could also act as a tumour suppressor in LUAD. This finding sheds light on how YY1 acts as both transcriptional activator and repressor. However, the specific mechanism of how YY1 acts as a bifunctional transcription factor needs further exploration.

A growing number of studies have indicated that lncRNAs are related to poor survival and that they have a strong potential to be novel biomarkers and targets for cancer treatment^24^. For example, PCA3, PCAT-1, PCGEM, and PCAT-18 are being investigated as potential diagnostic markers for prostate cancer^25,26^, while HOTRIA, AFAS1, and others are being considered as biomarkers for breast cancer^24,27^. Our results of ISH assay revealed that UPLA1 is associated with the TNM stage, N stage, and T stage. Univariate analysis indicated that UPLA1 could be an independent prognosis factor such as age and stage of LUAD in patients.

In conclusion, we identified a novel lncRNA UPLA1 that facilitated the progression of LUAD both in vivo and in vitro, promoted Wnt/β-catenin signalling by binding to DSP, and was found to be inversely regulated by YY1 transcription factor. UPLA1 may be used to predict poor prognosis in clinical settings. However, the interactions of lncRNA UPLA1 and other RNA-binding proteins and YY1 acted as both transcription activator and inhibitor need to be further explored. Further, an investigation of the potential therapeutic target of lncRNA UPLA1 is important. The exploration of carcinogenic lncRNAs using the CRISPR-Cas9 system will provide more insights into cancer therapy^28,29^.

## Supplementary information

Supplementary Figure

Supplementary Figure Legend

Supplementary Table
