# Supplementary figures and images for: Novel lncRNA UPLA1 mediates tumorigenesis and prognosis in lung adenocarcinoma
Source: Cell Death Dis. 2020 Nov 21;11(11):999. doi: 10.1038/s41419-020-03198-y (PMC7680460; doi:10.1038/s41419-020-03198-y)

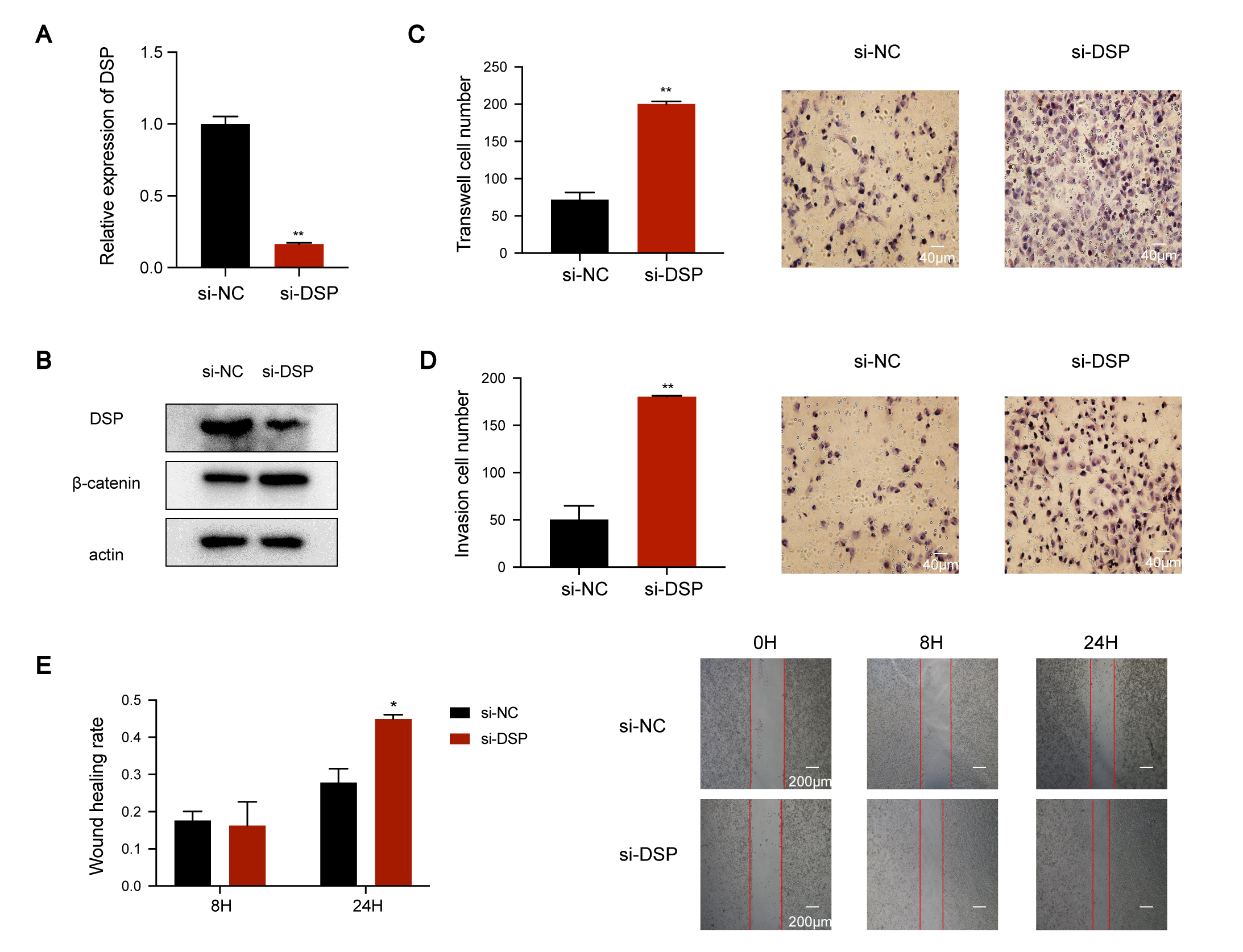

Supplement: Supplementary file 1 — Supplementary Figure [file 41419_2020_3198_MOESM1_ESM.tif]
