# Supplementary material for: Novel lncRNA UPLA1 mediates tumorigenesis and prognosis in lung adenocarcinoma
Source: Cell Death Dis. 2020 Nov 21;11(11):999. doi: 10.1038/s41419-020-03198-y (PMC7680460; doi:10.1038/s41419-020-03198-y)
Supplement: Supplementary file 2 — Supplementary Figure Legend [file 41419_2020_3198_MOESM2_ESM.docx]

**Supplementary Figure. DSP inhibits LUAD cells.** (A, B) Transfection efficacy was assessed by qRT-PCR and WB, which showed that β-catenin was up-regulated after inhibition of DSP. (C, D) Transwell and invasion assays demonstrated that the down-regulation of DSP promoted the metastatic and invasive abilities of the cells. (E) Wound healing assay revealed that the migration capability increased significantly after down-regulation of DSP. All values are expressed as mean ± SD (**P*<0.05, ***P*<0.01 by *t*-test).
