# Supplementary material for: Novel lncRNA UPLA1 mediates tumorigenesis and prognosis in lung adenocarcinoma
Source: Cell Death Dis. 2020 Nov 21;11(11):999. doi: 10.1038/s41419-020-03198-y (PMC7680460; doi:10.1038/s41419-020-03198-y)
Supplement: Supplementary file 3 — Supplementary Table [file 41419_2020_3198_MOESM3_ESM.docx]

Supplementary Table. Univariate and multivariate analyses of the factors correlated with Overall survival of Lung carcinoma patients

**Variables in the Equation**

| variables | Univariate analysis | | |  | Multivariate analysis | | |  |  |
| --- | --- | --- | --- | --- | --- | --- | --- | --- | --- |
|  | | HR | 95%CI | p value |  | HR | 95%CI | p value |  |
| UPLA1 expression | | 1.962 | 1.139-3.382 | 0.015 |  | 0.991 | 0.534-1.839 | 0.976 |  |
| sex | | 1.171 | 0.701-1.958 | 0.546 |  |  |  |  |  |
| Grade | | 1.008 | 0.642-1.582 | 0.973 |  |  |  |  |  |
| Age | | 1.026 | 1.002-1.051 | 0.036 |  | 1.015 | 0.987-1.043 | 0.295 |  |
| T stage | | 1.662 | 1.196-2.310 | 0.002 |  | 1.058 | 0.698-1.603 | 0.792 |  |
| N stage | | 2.976 | 1.691-5.238 | 0.000 |  | 1.710 | 0.874-3.345 | 0.117 |  |
| M stage | | 1.198 | 0.165-8.677 | 0.858 |  |  |  |  |  |
| TNM stage | | 2.160 | 1.587-2.939 | 0.000 |  | 1.834 | 1.141-2.946 | 0.012 |  |

* Statistically significant(p<0.05)
